# Characteristics of alveolar macrophages in bronchioalveolar lavage fluids from active tuberculosis patients identified by single-cell RNA sequencing

**DOI:** 10.7555/JBR.36.20220007

**Published:** 2022-05-28

**Authors:** Qianqian Chen, Chunmei Hu, Wei Lu, Tianxing Hang, Yan Shao, Cheng Chen, Yanli Wang, Nan Li, Linling Jin, Wei Wu, Hong Wang, Xiaoning Zeng, Weiping Xie

**Affiliations:** 1 Department of Respiratory and Critical Care Medicine, the First Affiliated Hospital of Nanjing Medical University, Nanjing, Jiangsu 210029, China; 2 Department of Tuberculosis, the Second Hospital of Nanjing, Nanjing, Jiangsu 210029, China; 3 Jiangsu Provincial Center for Disease Control and Prevention, Nanjing, Jiangsu 210029, China; 4 Department of Bioinformatics, Nanjing Medical University, Nanjing, Jiangsu 210029, China; 5 School of Biological Science and Medical Engineering, Southeast University, Nanjing, Jiangsu 210029, China

**Keywords:** tuberculosis, macrophage, bronchioalveolar lavage fluid, single-cell RNA sequencing

## Abstract

Tuberculosis (TB), is an infectious disease caused by *Mycobacterium tuberculosis* (*M. tuberculosis*), and presents with high morbidity and mortality. Alveolar macrophages play an important role in TB pathogenesis although there is heterogeneity and functional plasticity. This study aimed to show the characteristics of alveolar macrophages from bronchioalveolar lavage fluid (BALF) in active TB patients. Single-cell RNA sequencing (scRNA-seq) was performed on BALF cells from three patients with active TB and additional scRNA-seq data from three healthy adults were established as controls. Transcriptional profiles were analyzed and compared by differential geneexpression and functional enrichment analysis. We applied pseudo-temporal trajectory analysis to investigate correlations and heterogeneity within alveolar macrophage subclusters. Alveolar macrophages from active TB patients at the single-cell resolution are described. We found that TB patients have higher cellular percentages in five macrophage subclusters. Alveolar macrophage subclusters with increased percentages were involved in inflammatory signaling pathways as well as the basic macrophage functions. The TB-increased alveolar macrophage subclusters might be derived from M1-like polarization state, before switching to an M2-like polarization state with the development of*M. tuberculosis* infection. Cell-cell communications of alveolar macrophages also increased and enhanced in active TB patients. Overall, our study demonstrated the characteristics of alveolar macrophages from BALF in active TB patients by using scRNA-seq.

## Introduction

Tuberculosis (TB) is an infectious disease caused by*Mycobacterium tuberculosis* (*M. tuberculosis*) with a long history dating from at least 5000 years^[1–2]^. According to the newest Global WHO TB report released in 2021^[3]^, approximately 9.9 million people developed TB in 2020, including 1.2 million people who died from the disease. This makes TB one of the leading causes of death worldwide^[4]^. However, the mechanisms involved in *M. tuberculosis* interactions with our immune systems or TB-typically-affected organs like the lungs remain unclear^[5]^. This is one of the obstacles we face in finding new biomarkers for diagnosis and new targets to develop effective interventions or innovative vaccines to prevent the spread of severe TB symptoms^[6]^.

As the primary immune cells that respond first to *M. tuberculosis* infections in the lung alveolar spaces, alveolar macrophages are known to contribute to the formation or maintenance of TB-granuloma and are believed to regulate adaptive immune responses by producing various chemokines and cytokines during infection^[7–8]^. With evidence of alveolar macrophage heterogeneity and functional plasticity emerging in recent years, the prognosis of TB has proven to be associated with different macrophage phenotypes and different activations and polarization statuses^[8]^. Traditionally, the macrophage polarization status has been divided into two clusters including M1 macrophages (*i.e.*, classically activated macrophages) and M2 macrophages which are alternately activated macrophages^[9]^. Given the different gene markers expressed and distinct chemokines or cytokines secreted, M1 macrophages are usually considered as pro-inflammatory macrophages associated with antimicrobial immunity, while M2 macrophages are considered anti-inflammatory macrophages associated with immunosuppression^[10]^. However, recent *in vivo* studies have suggested that alveolar macrophages from TB modeled mice and human monocyte-derived macrophages with TB infections, express increased M1-polarized markers^[11–12]^. During TB infection, different phenotypes or polarization states of macrophages might affect the growth of bacilli in a restrictive or permissive manner^[8]^. Therefore, it is reasonable to suppose that TB infections influence the progress of macrophage polarization. In fact, alveolar macrophages could be divided into several subclusters according to their transcriptional profiles in healthy individuals and there was a small population of pro-inflammatory or M1-like alveolar macrophages in bronchioalveolar lavage fluid (BALF) cells of healthy adults^[13]^.

However, the proportion or function of the various alveolar macrophages subclusters from TB patients are different from healthy controls remains unclear, which need to be studied thoroughly. To date, many studies have been based on tissue or blood RNA sequencing from patients with TB and have provided new insights into the pathogenesis of TB^[14–15]^. While only specific cell types of interest can be detected by flow cytometry, heterogenicity can be concealed within average measures observed using bulk RNA sequencing. Presently, cell-to-cell variation and their interactions can be identified with an unprecedented single-cell resolution, and with the development of single-cell RNA sequencing (scRNA-seq)^[16]^. Currently, several studies which applied scRNA-seq to peripheral blood mononuclear cells of TB patients or granuloma tissues from *M. tuberculosis*-infected animal models^[17–19]^. Given that respiratory pathways are the main portals of entry for *M. tuberculosis*, we need more pulmonary specific studies focusing on alveolar macrophages from BALF cells than we do studies of peripheral blood or studies using animal modeling^[20]^, especially at single-cell resolution. To date, no studies have been published around the single-cell landscape of the alveolar macrophages from active TB patients. In this study, we performed scRNA-seq analysis to characterize alveolar macrophages from BALF in TB patients and made a comparison of BALF samples with healthy controls. We believe this will help uncover the mechanisms of host defense against *M. tuberculosis* and in the discovery of novel vaccines or therapeutic targets as there are an increasing number of host-directed interventions which target macrophages^[21–22]^.

## Materials and methods

We collected BALF samples from three patients with active TB hospitalized in the Second Hospital of Nanjing in January and February of 2021. Our study was approved by the Ethics Committee of the First Affiliated Hospital of Nanjing Medical University (No. 2020-SRFA-339). We conducted this study in accordance with ethical standards established in the Helsinki Declaration.

Demographics and clinical characteristics of the participants have been provided in ***Supplementary Table 1*** (available online). All participants were newly-diagnosed, bacteriologically confirmed pulmonary TB patients according to positive smear microscopy, positive *M. tuberculosis* cultures and positive GeneXpert MTB/RIF in their sputum or BALF according to the WHO revised definitions for active TB^[23]^. The bacterial strains of *M. tuberculosis* isolated from the patients were sensitive to isoniazid and rifampicin. The three TB patients had no history of other chronic diseases such as cancer, human immunodeficiency virus infection and autoimmune diseases. The patients in this sample underwent fiberoptic bronchoscopy before receiving anti-TB treatment. A total of 10 mL BALF were collected from each patient and kept on ice immediately after a fiberoptic bronchoscopy was performed within 2 hours. All BALF samples were processed in a BSL-3 laboratory.

### Isolation of single cell and single-cell RNA sequencing

The BALF was filtered by a 100-μm cell strainer (Biosharp, China) before being centrifuged at 800 *g* for 5 minutes. Then, we used 3 mL red blood cell lysis buffer (Beyotime, China) to resuspend cell pellets after removing the supernatant and incubating these cells for 3 minutes. After being centrifuged at 800 *g* for 5 minutes, the supernatant was removed and the precipitate was resuspended at 1×10^6^ cells/mL in a cooled Dulbecco's phosphate saline buffer (Servicebio, China) containing 0.05% bovine serum albumin (Beyotime). We stained BALF cells with trypan blue (Invitrogen, USA) and determined cellular viability with an automated cell counter (Invitrogen) to ensure cell viability was over 90% in each sample. We used the Chromium Next GEM Single Cell 3ʹ Gel Bead Kit V3.1 (10× Genomics, USA) to perform single-cell capture and library construction in accordance with the manufacturer's instructions. Constructed sequencing libraries were sequenced with the Illumina sequencer (Illumina, USA).

### Pre-processing of scRNA-seq data and quality control

We conducted data de-multiplexing, gene expression quantification of unique molecular identifier counts and alignment to the GRCh38 human genome using Cell Ranger Software (version 3.1.0, 10× Genomics). All raw data of sequencing were uploaded and available in the GSA for Human data repository of the China National Center for Bioinformation (HRA001418). Cells expressing less than 200 genes or more than 10% of mitochondrial gene reads, were ruled out of further analysis. We also removed cells with less than 1% of ribosomal genes or over 5% of hemoglobin genes from this study. To attenuate batch effects on the scRNA-seq data, we performed canonical correlation analysis from the Seurat R package (version 4.0.3)^[24]^. All codes used in this study have been presented in the supplementary file named "code_for_use.pdf" (available online). BALF scRNA-seq data of healthy controls (*i.e.*, GSM4475048, GSM4475049, and GSM4475050) were obtained from the Gene Expression Omnibus (GEO) database (GSE145926)^[25]^.

### Data integration, dimensionality reduction and clustering

scRNA-seq data were integrated from all samples including TB patients and healthy controls after controlling for the batch-effect, based on the top 2000 most informative genes defined by Seurat's FindVariableFeatures function with the Seurat R package (version 4.0.3)^[26]^. Dimensionality reduction of the integrated data was conducted using Uniform Manifold Approximation and Projection (UMAP)^[27]^. We clustered and visualized integrated scRNA-seq data in this study with the top 30 principal components by using the Seurat R package (version 4.0.3) at the resolution of 0.8. The same aforementioned protocols were followed when we re-integrated and re-clustered alveolar macrophages at the resolution of 0.1. We performed differential gene expression (DGE) analysis by the method called Model-based Analysis of Singlecell Transcriptomics (Seurat's FindAllMarkers function)^[28]^. We identified the cluster markers for each alveolar macrophage subcluster with DGE analysis by comparing one alveolar macrophage subcluster with other subclusters. Cluster markers were defined as differentially expressed genes (DEGs) with log_2_(fold changes)>0.25 or <−0.25 and adjusted*P*-values<0.05. We considered DEGs with log_2_(fold changes)>0 as upregulated DEGs, while DEGs with log_2_(fold changes)<0 were considered, downregulated DEGs.

### Functional enrichment analysis and pseudo-temporal trajectory analysis

We performed functional enrichment analysis based on the Kyoto Encyclopedia of Genes and Genomes (KEGG) database^[29]^ using web-accessible, functional annotation tools, and the Database for Annotation, Visualization and Integrated Discovery tools^[30]^. Detailed results from all KEGG analyses have been provided in the supplementary materials. Pseudo-temporal trajectory analysis was conducted using Monocle 3 software^[31]^ with pseudo-temporal trajectories visualized using the SimplePPT algorithm^[32]^ within Monocle 3 software after a dimensionality reduction process. Pseudo-times for every single cell were defined according to geodesic distances to the trajectory branch root. This was established according to specific biological functions.

### Cell-cell communication analysis

Intercellular communication analysis was conducted using different BALF cell types, which were visualized by applying the CellChat tool^[33]^. Multilayer signal networks for different macrophage subclusters were constructed using a method first presented by Zhang *et al*^[34]^ and Cheng*et al*^[35]^. This method requires ligands, receptors, transcriptional factors (TF) and their target genes. Information regarding ligand-receptor pairs, receptor-TF connections and TF-target-gene pairs were extracted from Transcriptional Regulatory Element Database^[36]^, the KEGG database^[29]^, Search Tool for the Retrieval of Interacting Genes/Proteins database^[37]^, OmniPath database^[38]^ and other previously published studies^[34–35]^. The multilayer signal network was visualized and characterized using Cytoscape^[39]^.

### Statistical analysis

The percentages related to different macrophage subclusters between those with active TB and healthy controls were analyzed using an unpaired version of Student's *t*-test (two-tailed,) with GraphPad Prism software (version 8.4.0). The non-parametric Wilcoxon rank sum test was used for DGE analysis in Seurat R package (version 4.0.3). *P*-values of less than 0.05 were considered statistically significant.

## Results

### Landscape of BALF cells from TB patients and healthy controls

**Figure 1:**
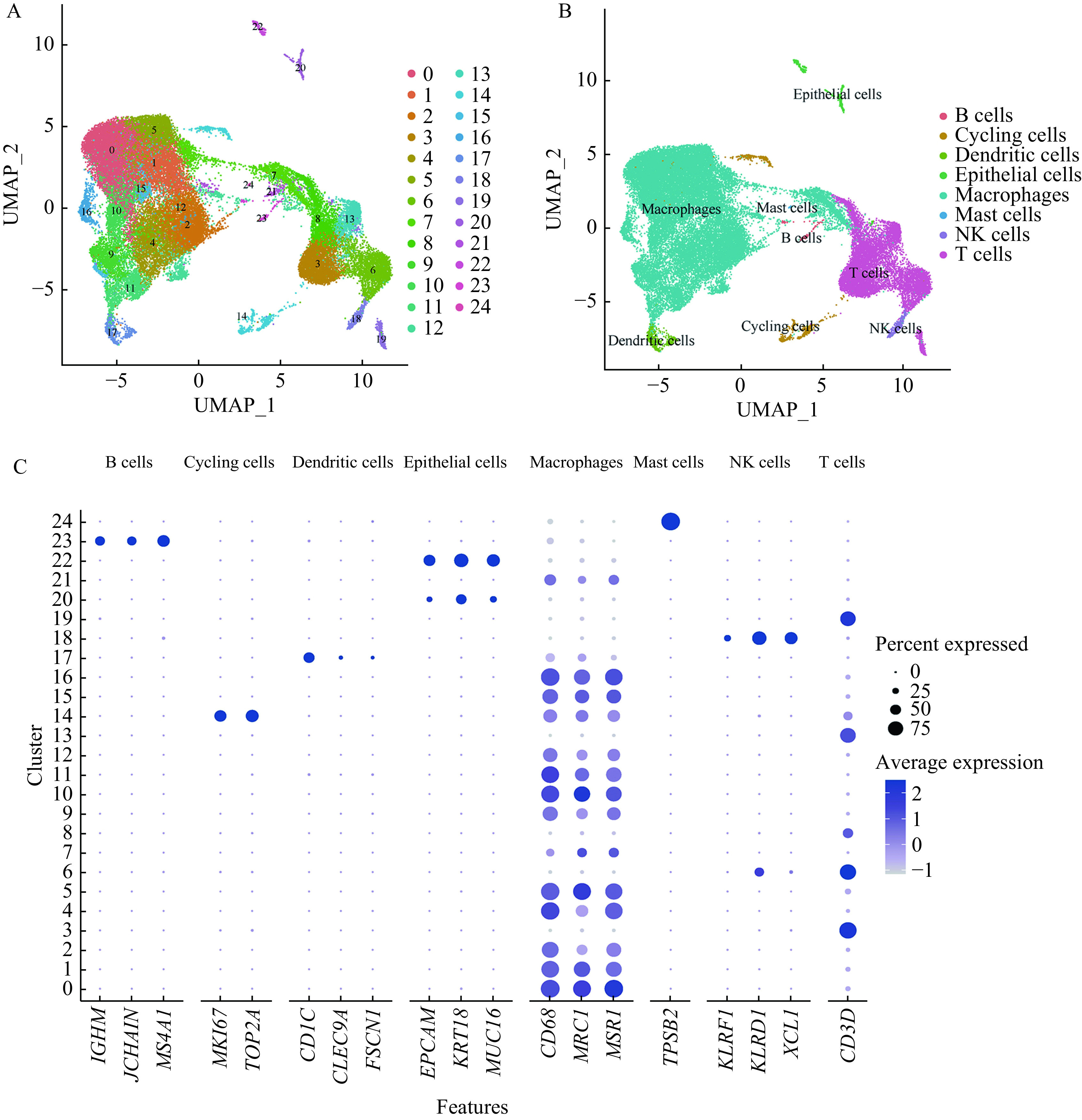
Single cell transcriptional profiling of BALF cells.

**Figure 2:**
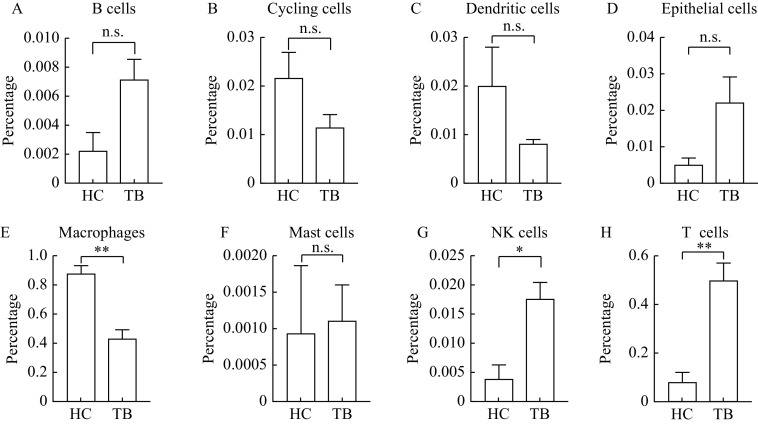
Comparisons of the proportions of the 8 different BALF cell types between healthy controls and tuberculosis patients.

We performed 10× Genomics Chromium droplet scRNA-seq to characterize a total of the 16 655 BALF cell collected from three active TB patients (one female and two males) after applying quality control protocols mentioned in the methods section. We then compared BALF cells from active TB with healthy controls by analyzing scRNA-seq BALF cell data from three healthy adults in the GEO database (GSE145926), simultaneously. A total of 25 clusters in BALF cells were identified using cluster analysis. Please see ***Fig. 1A*** for further details. We also provided a demonstration of single BALF cell transcriptional profiling, from both our TB patients and healthy controls (***Supplementary Fig. 1***, available online). Eight cell types were annotated with transcriptional cell markers (***Table 1***), used in previously published research^[13,25,40]^. This included B cells (cluster 23), cycling cells (cluster 14), dendritic cells (cluster 17), epithelial cells (clusters 20, 22), macrophages (clusters 0–2, 4–5, 7, 9–12, 15–16, 21), mastocytic cells (cluster 24), natural killer (NK) cells (cluster 18), T cells (clusters 3, 6, 8, 13, 19). Please see ***Fig. 1B*** and ***C*** for further information. We found that the percentage of macrophages decreased significantly (*P=*0.006) in active TB patients compared to healthy controls (***Fig. 2***).

**Table 1:** Cell type annotations and the gene marker(s) of each type used in ***Fig. 1***

Cell type annotations	Cluster number	Gene markers
B cells	23	*IGHM, JCHAIN, MS4A1*
Cycling cells	14	*MKI67, TOP2A*
Dendritic cells	17	*CD1C, CLEC9A, FSCN1*
Epithelial cells	20, 22	*EPCAM, KRT18, MUC16*
Macrophages	0–2, 4–5, 7, 9–12, 15–16, 21	*CD68, MRC1, MSR1*
Mast cells	24	*TPSB2*
Natural killer cells	18	*KLRF1, KLRD1, XCL1*
T cells	3, 6, 8, 13, 19	*CD3D*

### Various alveolar macrophage subclusters identified by DGE analysis

**Figure 3:**
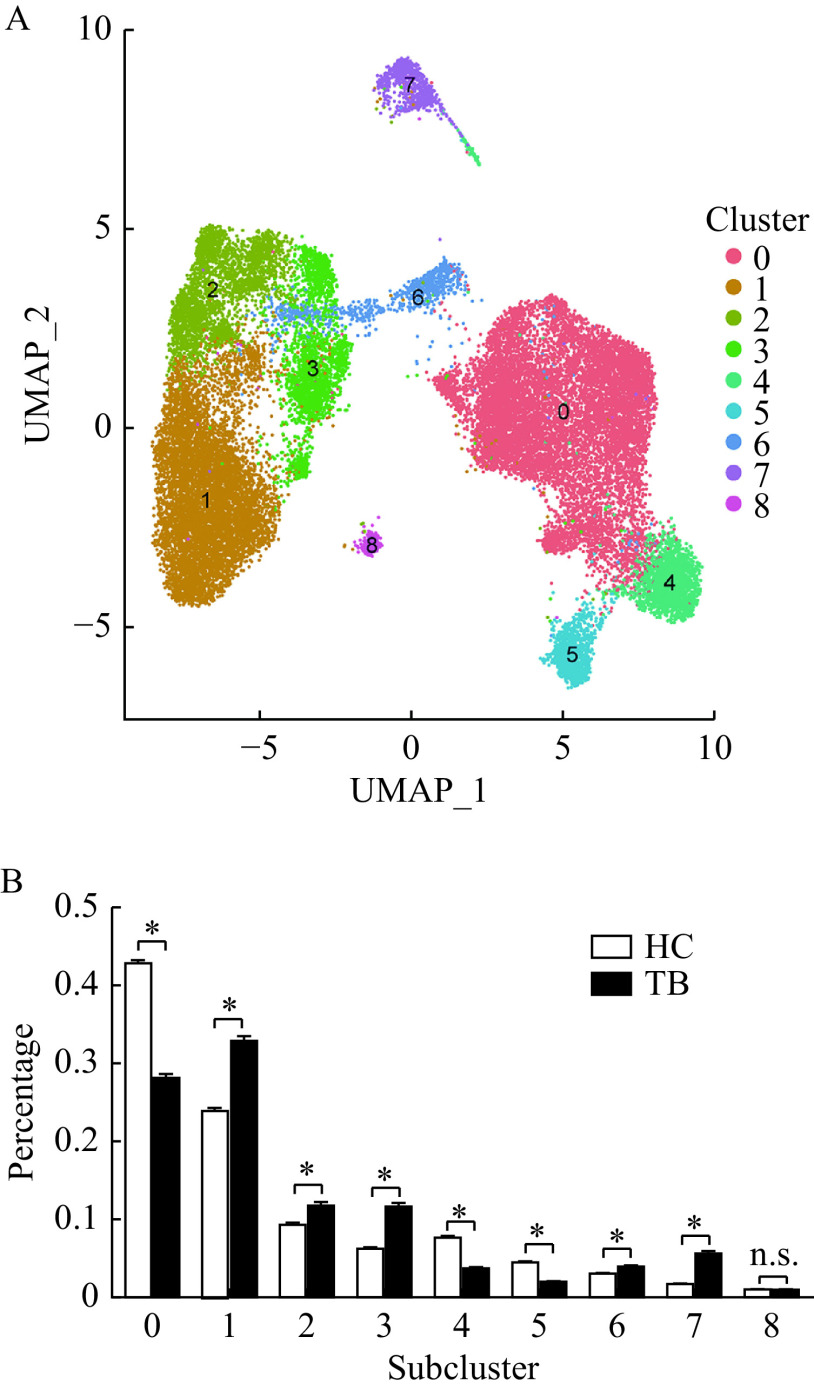
Different subclusters of alveolar macrophages identified in BALF cells.

Since macrophage populations comprise the largest proportion of BALF cells which significantly decreases in active TB patients, we re-clustered macrophages to further analyze the potential heterogeneity which may have been concealed by general cell types. We identified 9 subclusters of alveolar macrophage in BALF cells (***Fig. 3A***) with each subcluster comprised of cells from both active TB patients and healthy controls (***Fig. 3B***). We also found that these alveolar macrophages from BALF cells were distributed differently across our TB patient sample and healthy controls, after removing the batch effect of each sample.

**Figure 4:**
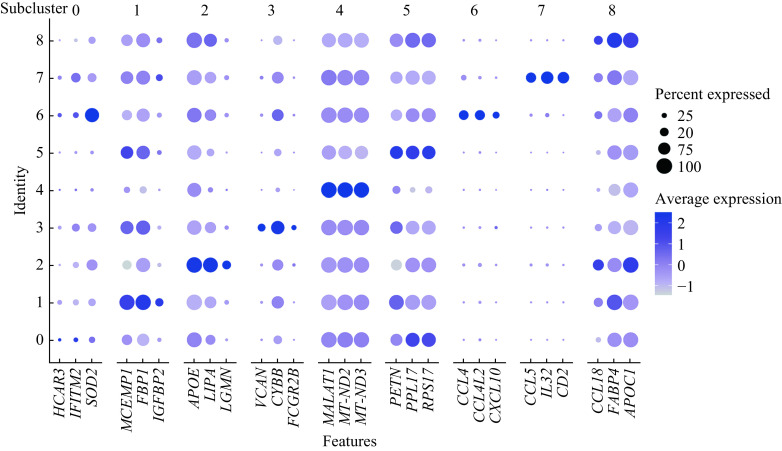
Dot plot showing the expression of the top 3 marker genes in each macrophage subcluster across all 9 macrophage subclusters.

Active TB patients had higher percentages of alveolar macrophages in subclusters 1–3, 6, and 7 compared to healthy controls (***Fig. 3B***). Although, they had lower percentages of alveolar macrophages in subclusters 0, 4, and 5. No differences existed in subcluster 8 between the two groups (***Fig. 3B***). DGE analysis was conducted to understand gene markers (***Table 2***) for all 9 alveolar macrophage subclusters and their unique functions can be seen in ***Fig. 4***. We found that several alveolar macrophage subclusters, including subclusters 4, 5, and 8, expressed slightly different marker genes across all macrophage subclusters, which might be a result of transcriptional spectrum from macrophages.

**Table 2:** Gene markers of the 9 alveolar macrophage subclusters used in ***Fig. 4*** revealed by differential gene expression analysis

Alveolar macrophage subclusters	Gene markers
Subcluster 0	*HCAR3, IFITM2, SOD2*
Subcluster 1	*MCEMP1, FBP1, IGFBP2*
Subcluster 2	*APOE, LIPA, LGMN*
Subcluster 3	*VCAN, CYBB, FCGR2B*
Subcluster 4	*MALAT1, MT-ND2, MT-ND3*
Subcluster 5	*RETN, RPL17, RPS17*
Subcluster 6	*CCL4, CCL4L2, CXCL10*
Subcluster 7	*CCL5, IL32, CD2*
Subcluster 8	*CCL18, FABP4, APOC1*

There were two alveolar macrophage subclusters (subclusters 6 and 7) with discrete marker genes from other subclusters, which might imply distinct functions from other subclusters. Then, we performed a functional enrichment analysis to explore the potential biological functions of these different alveolar macrophage subgroups.

### Functional enrichment analysis of all alveolar macrophage subclusters

**Figure 5:**
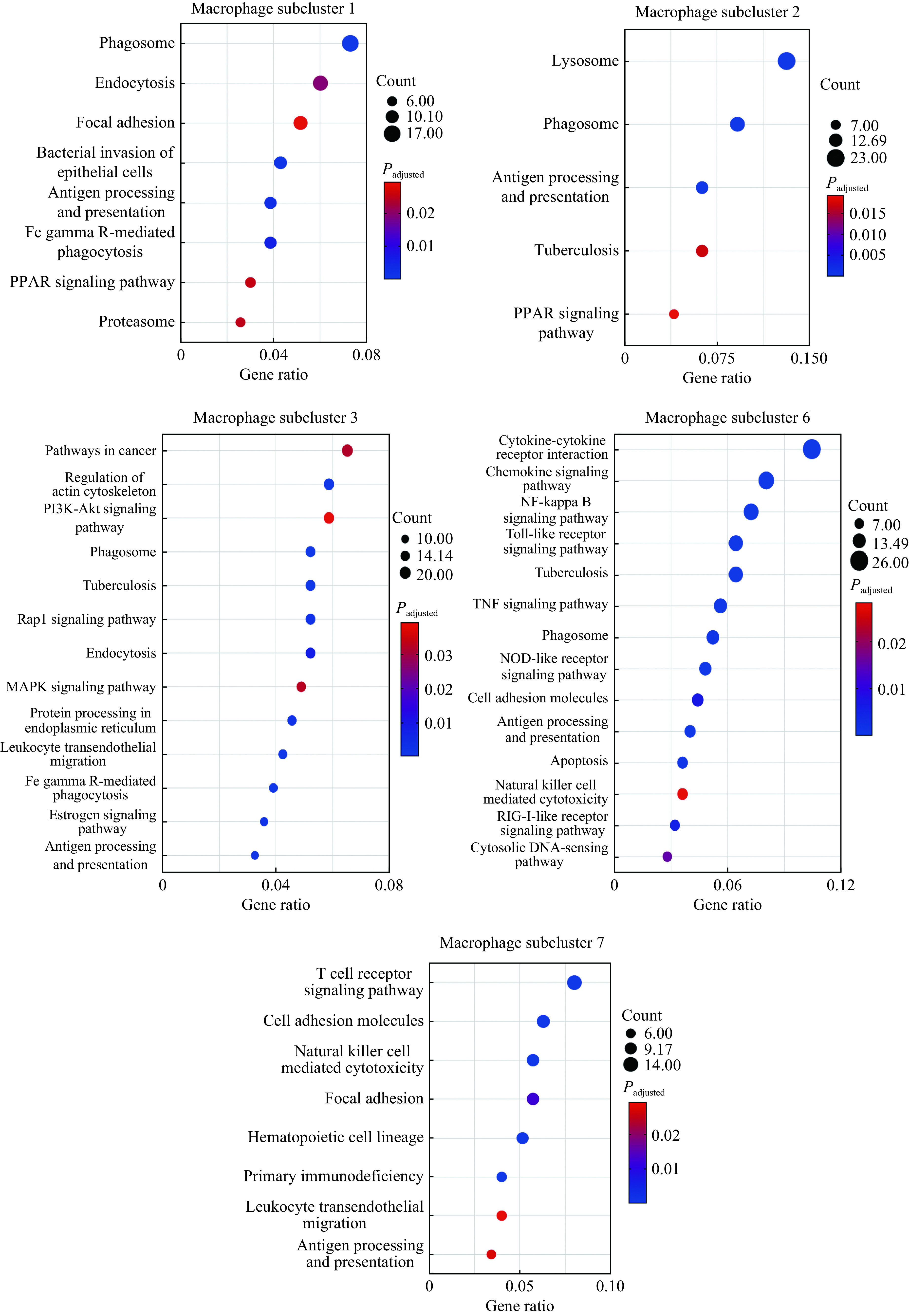
Functional enrichment analysis of macrophage subclusters that increased in active tuberculosis patients (subclusters 1–3, 6, and 7) with the upregulated differentially expressed genes.

Functional enrichment analysis of all alveolar macrophage subclusters (subclusters 1–3, 6, and 7) that significantly increased in active TB patients with the upregulated DEGs are provided in ***Fig. 5***. KEGG analysis demonstrated that the macrophage subclusters (subclusters 1–3, 6, and 7), which increased in active TB patients, were involved in inflammatory signaling pathways such as the PPAR signaling pathway, TNF pathway, NF-kappa B pathway, chemokine signaling, and the Toll-like receptor signaling pathway. These are in addition to the basic functions of macrophage which include antigen processing and presentation, phagocytosis, cell adhesion and endocytosis. At the same time, upregulated DEGs in TB-related pathways were significantly enriched in subcluster 2 (adjusted *P*=0.017), subcluster 3 (adjusted *P*<0.001) and subcluster 6 (adjusted*P*<0.001). See***Table 3*** for further details.

**Table 3:** Enriched TB-related pathways of macrophage subclusters by KEGG analysis

Subclusters	2	3	6
Enriched genes of TB-related pathways	*HLA-DMA, HLA-DMB, LAMP1, STAT1, ITGB2, CALM3, CD14, CTSD, ATP6V0D2, CTSS, HLA-DQA1*	*CIITA, CEBPB, TGFB1, ITGAM, STAT1, IFNGR2, ITGB2, CORO1A, HSPD1, FCGR3A, FCGR2A, MRC1, CD14, CALM1, FCGR2B, JAK1*	*FCER1G, STAT1, RIPK2, IFNGR2, IL18, TNF, CTSS, NFKB1, IL1A, IL1B, CD14, FCGR1A, CLEC4E, BID, HLA-DQA1, TLR2*
Count	11	16	16
Gene ratio	0.063	0.052	0.072
False discovery rate	0.016	<0.001	<0.001
Adjusted *P*-value	0.017	<0.001	<0.001
TB: tuberculosis; KEGG: Kyoto Encyclopedia of Genes and Genomes.			

From the result of the KEGG analysis, we found that subclusters 6 and 7 were indeed more likely to have special biological functions with specific DEGs, distinct from other alveolar macrophage subclusters. The two most enriched pathways in subcluster 6 were cytokine-cytokine receptor interactions and the chemokine signaling pathway. It is reasonable to assume that subcluster 6 is likely to produce or interact with cytokines and chemokines. We noted that the subcluster 7 was also associated with the T cell receptor signaling pathway and NK cell mediated cytotoxicity signaling as well as those signaling pathways mentioned previously. These indicate the potential interactions within this subcluster in terms of cell-mediated immunity, including T and NK cells.

### Polarization states of all alveolar macrophage subclusters

**Figure 6:**
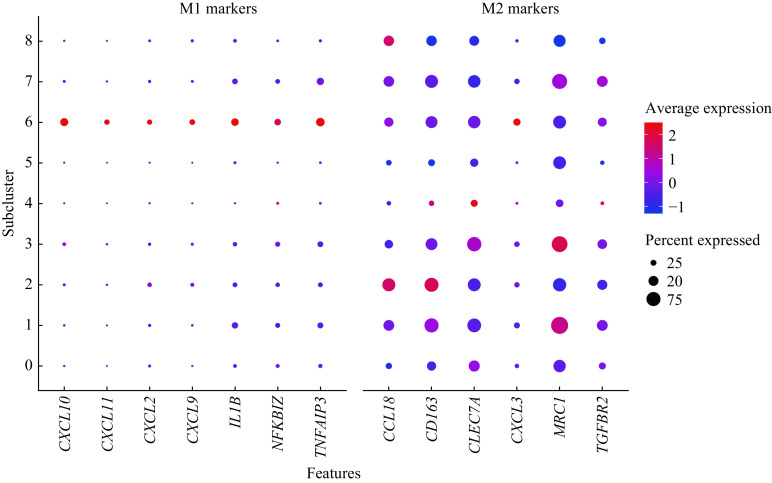
Dot plot of M1-like and M2-like macrophage gene markers expression across the 9 macrophage subclusters.

We next identified polarization states for each alveolar macrophage subcluster by analyzing transcriptional profiles. This was done because functional enrichment analysis revealed that all increased alveolar macrophage subclusters in active TB patients were related to inflammation-related signaling. We compared DEGs of all 9 subclusters with gene marker panels related to M1 and M2 macrophages, which were referred to by other researchers^[9–10,41]^. The results of the DGE analysis indicated that the alveolar macrophage subclusters 6 exhibited enhanced expression of both M1-like and M2-like macrophage gene markers (***Fig. 6***). Although, we also found that alveolar macrophage subclusters 0, 4, and 5 had lower expression of M2 macrophage gene markers. However, this was without any significant expression of M1-like macrophage gene markers compared to other alveolar macrophage subclusters. It is therefore reasonable to assume that alveolar macrophage subclusters 0, 4, and 5 might be M0 macrophages, without M1 or M2 activation. Moreover, alveolar macrophage subclusters 0, 4, and 5 formed the main alveolar macrophage populations which decreased in active TB patients compared to healthy controls.

In addition, the result of the DGE analysis indicated that all of the five TB-increased alveolar macrophage subclusters, including subclusters 1–3, 6, and 7, exhibited higher expression of M2-like macrophage gene markers. Therefore, we assumed that alveolar macrophage subclusters 1–3 and 7 were M2-like macrophages and that subcluster 6 might be considered as a special population with coexistence of both M1-like and M2-like polarization states. These results concerned with macrophage polarization states described supports the notion that there were no independent M1-like macrophages with only enhanced M1-like gene markers. Although, these are likely to be without any enhanced M2-like gene markers in any macrophage subclusters in active TB patients. This is consistent with other scRNA-seq studies focusing on resident macrophages located in human lung cancer tissues^[42]^.

### Relationship between alveolar macrophage subclusters

**Figure 7:**
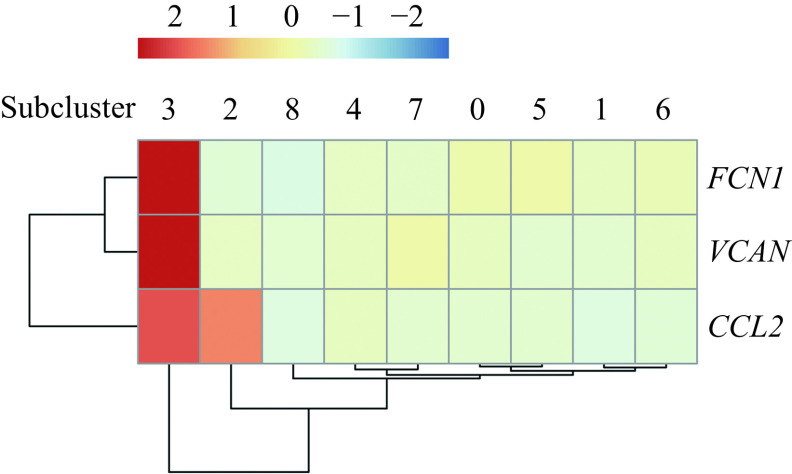
Expression of the monocyte-like macrophage gene markers across the 9 macrophage subclusters.

**Figure 8:**
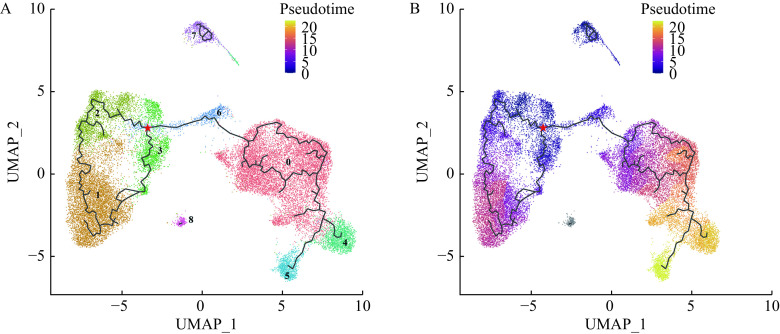
UMAP plot of different macrophage subclusters colored by pseudo-temporal trajectory analysis.

Trajectory analysis of alveolar macrophage subclusters was conducted to explore possible associations between subclusters. In accordance with a previous study^[13]^, we applied three gene markers including *FCN1*, *VCAN*, and *CCL2* to identify the monocyte-like macrophages within the airspace. In ***Fig. 7***, you can see that alveolar macrophage subcluster 3 was the *FCN1*, *VCAN*, and *CCL2* positive cell subcluster which can be considered the monocyte-like macrophages within the airspace. Since alveolar macrophages are thought to originate from monocyte-like macrophages^[13]^, we considered the alveolar macrophage subcluster 3 with high expressions of *FCN1*, *VCAN*, and *CCL2* as the root of the pseudo-temporal trajectory branch. This comprised of all alveolar macrophage subclusters and can be seen in ***Fig. 8A***. From the pseudo-temporal trajectory branch (***Fig. 8B***), we found that the position of macrophages from the subclusters 2 and 3 was followed by subcluster 6 among the five increased macrophage subclusters (1–3, 6, and 7) in TB patients. Although, subcluster 1 followed subcluster 6 along the pseudo-temporal trajectory branch. The alveolar macrophage subclusters 1–3 were considered M2-like alveolar macrophages and alveolar macrophage subcluster 6 expressed higher M1-like marker genes as well as M2-like marker genes. Therefore, it was reasonable to assume that pseudo-temporal trajectory analysis represented changes of polarization states from M1-like polarization state to M2-like polarization state in the alveolar macrophages which increased in active TB patients.

### Intercellular communication and multilayer signal networks

**Figure 9:**
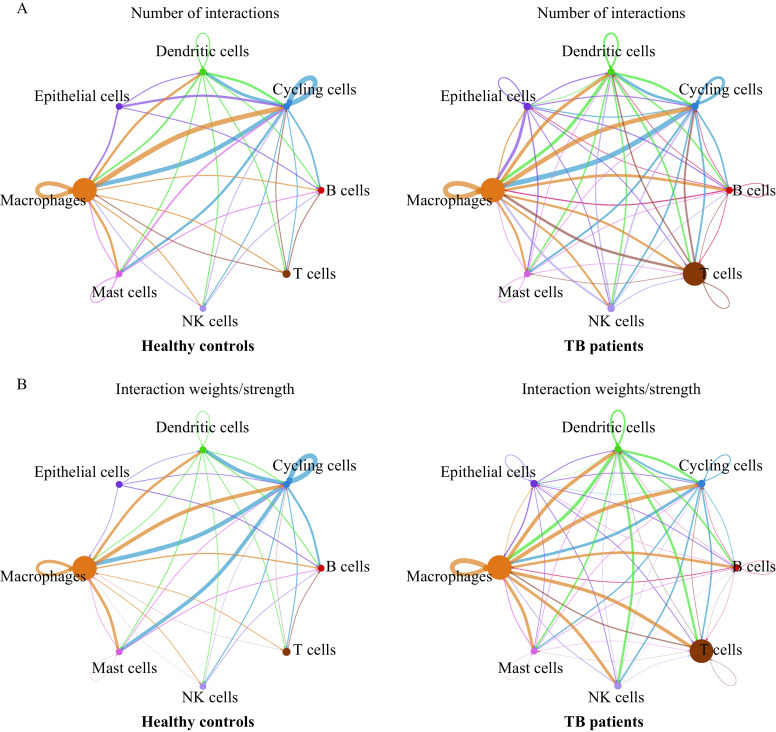
Intercellular communications between macrophages and other cell types in tuberculosis patients and healthy controls.

We analyzed ligand-receptor communications between macrophages and other cell types by CellChat (***Fig. 9***) including TB patients and healthy controls. We found that there were more intercellular communications between macrophages and other cell types in TB patients in ***Fig. 9A***. New macrophage interactions emerged in our sample of TB patients including connections to epithelial cells and connections to B cells. This suggests there is an effect of epithelia and B cells on macrophages in TB patients. Additionally, communications between macrophages and T cells or NK cells were enhanced in TB patients (***Fig. 9B***). As well as an increased number of intercellular communications, the number of interactions between different macrophage subclusters and their interaction weights were also increased in TB patients (***Fig. 9***). Therefore, we conducted multilayer signal network analysis between macrophage subclusters.

**Figure 10:**
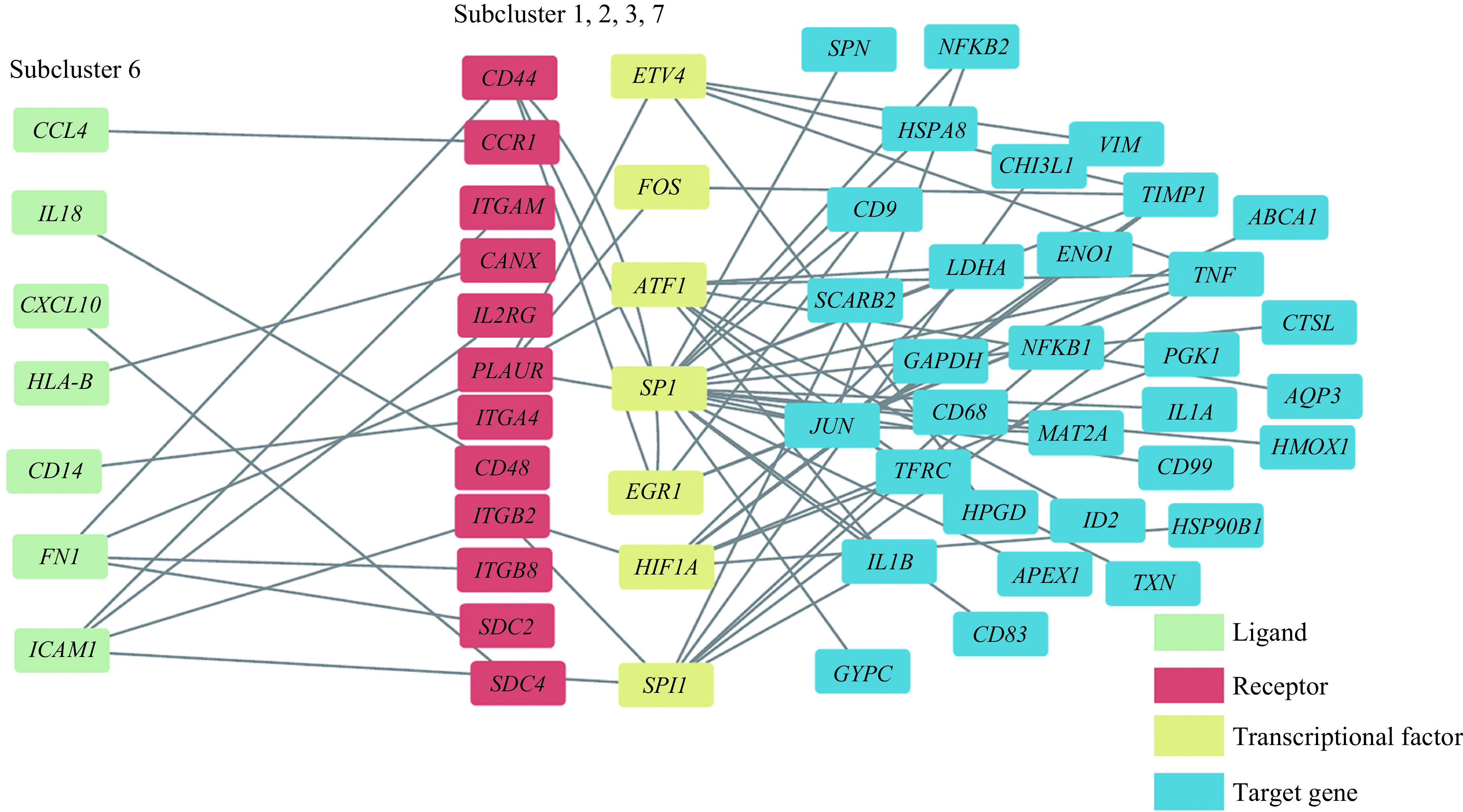
Multilayer intercellular and intracellular signal networks among different macrophage subclusters.

Based on the results of functional enrichment analysis described above, the macrophage subcluster 6 was related to cytokine-cytokine receptor interaction pathway and chemokine signaling pathway. Then, we constructed a multilayer signal networks of the TB-increased macrophage subcluster 1, 2, 3, and 7 in response to the cytokines or chemokines of subcluster 6 (***Fig. 10***). We found that there were some receptors related to TB-associated protective immunity^[43–44]^ or macrophage recruitment^[45]^ such as *CD44*, *CCR1*, and *IL2RG* in the multilayer signal networks. The appearance of a series of genes which encode integrin or syndecan including *ITGAM*, *ITGA4*, *ITGB8*, *SDC2*, and *SDC4* indicated that integrin or syndecan modulate the TB-induced immune response^[46–47]^. Additionally, *HIF1A* and *ATF1*, one of the TFs shown in ***Fig. 10*** were linked to immunometabolism^[48]^. Finally, some pro-inflammatory genes like *IL1B* and *TNF* were seen as target genes within the multilayer signal networks.

## Discussion

In this study, we characterized alveolar macrophages from BALF cells of active TB patients by using scRNA-seq and then grouped these into 9 subclusters, according to transcriptional profiles. We found that the proportions of five alveolar macrophage subclusters significantly increased in active TB patients compared with healthy controls. We also analyzed their presumptive biological functions as well as polarization states. Two novel alveolar macrophage subclusters were identified with distinct functions related to chemokines and adaptive immune response. Alveolar macrophages, as the largest population of the BALF cells, were considered a homogeneous population in a previous study^[49]^. However, a recent study demonstrated that the heterogeneity of alveolar macrophages existed in healthy adults by scRNA-seq^[13]^.

We also found that there were several subclusters of alveolar macrophages in active TB patients and healthy controls. The percentages of some specific alveolar macrophage subclusters also increased in active TB patients when compared to healthy controls. This suggests that these alveolar macrophage subclusters might play an important role in host defenses against *M. tuberculosis*. The result of functional enrichment analysis of these subclusters suggested that these TB-activated subclusters were related to inflammation and TB-associated signaling pathway. This finding was according to past research to be expected. Macrophage-mediated host defenses including antimicrobial immune response are mediated by macrophage polarization or activation^[9]^.

In this study, we identified a total of four macrophage subclusters as M2-like macrophages and a special subcluster as M1-like and M2-like macrophages that increased in TB patients. Although the result of this study seems to contradict the canonical dichotomous model of macrophage polarization with either M1-like (pro-inflammatory) macrophages or M2-like (anti-inflammatory) macrophages^[10]^. This can be explained by new evidence which suggests there are no independent M1-like macrophage subclusters but rather macrophage subclusters with M1-like and M2-like marker co-existence. This has been found in several human lung tissue and mice BALF cells^[42,50]^. For example, Mould *et al* concluded that alveolar macrophages, collected from BALF samples of healthy individuals, are matured from bone marrow-derived monocytes. Given that the monocytes of TB patient have a tendency to differentiate into M2-like macrophages^[51]^, our study re-inforces that all the alveolar macrophage subclusters with increased percentages in TB patients expressed enhanced M2-like gene markers, which supports the hypothesis proposed by Mould *et al.*

According to pseudo-temporal trajectory analysis conducted here, TB-increased alveolar macrophage subcluster with the coexistence of M1-like and M2-like macrophages which might be located between the root of pseudo-temporal trajectory branch and M2-like macrophage subclusters. This suggests that the development of *M. tuberculosis* infection results in alveolar macrophage polarization state alterations. In other *in vivo* and *in vitro* studies of TB, the alveolar macrophages collected from mice BALF exposed to *M. tuberculosis* or human monocyte-derived macrophages stimulated by TB-specific virulent factor shifted their polarization states from M1 to M2 when TB-induced inflammation increased^[11,41]^. The directions where the alveolar macrophages of our study shifted are consistent with the previous studies.

Through cell-cell communication analysis, we found that macrophage communications from BALF samples increased in TB patients. There may be some existing evidence which provides an explanation for this phenomenon. On one hand, the secretion of cytokines and chemokines of macrophages increases after inflammation induced by bacterial infection^[52–53]^. Yet from another, as signal transport vectors of cell communications, the exosomes produced by *M. tuberculosis* or macrophages may modulate the host-immune response in TB patients^[54–55]^. Overall, for a better understanding of TB pathogenesis in the human body, it is necessary to conduct further research including BALF samples of TB patients with different disease severity in the future.

We admit that there were a few limitations in our study. Even though transcriptional profiles of airspace macrophages were isolated from BALF cells and conserved across healthy adults of different ages or genders^[13]^, potential selection bias might exist because of the relatively small sample size of this study. Therefore, we made batch-effect corrections before performing further analysis of all scRNA-seq data in our study. Also, as all participants in this study were patients with pulmonary active TB, the characteristics of alveolar macrophages in latent TB or other TB subtypes were not investigated. This provides a number of new areas of research which must be conducted to enhance our understanding in this field.

In conclusion, we identified eight cell types in BALF cells at single-cell resolution. We also characterized alveolar macrophages from BALF in TB patients through DGE analysis and functional enrichment analysis. A total of four alveolar macrophage subclusters were identified as M2-like macrophages and a special subcluster as M1-like and M2-like macrophages among the alveolar macrophages that increased in TB patients. The alveolar macrophage subclusters that increased in TB patients might be derived through the M1-like polarization state. These then switch to an M2-like polarization state with the development of *M. tuberculosis* infection. Cell-cell communications from alveolar macrophages also increased and enhanced in active TB patients. To the best of our knowledge, no scRNA-seq data of BALF cells in TB patients have been published or analyzed previously. Our study may further the understanding of the role of alveolar macrophages in TB pathogenesis and will be of great value for exploring novel therapeutic targets against TB in the future.
